# Peroxidase-mediated mucin cross-linking drives pathologic mucus gel formation in IL-13–stimulated airway epithelial cells

**DOI:** 10.1172/jci.insight.181024

**Published:** 2024-06-18

**Authors:** Maude A. Liegeois, Margaret Braunreuther, Annabelle R. Charbit, Wilfred W. Raymond, Monica Tang, Prescott G. Woodruff, Stephanie A. Christenson, Mario Castro, Serpil C. Erzurum, Elliot Israel, Nizar N. Jarjour, Bruce D. Levy, Wendy C. Moore, Sally E. Wenzel, Gerald G. Fuller, John V. Fahy

**Affiliations:** 1 Cardiovascular Research Institute, University of California, San Francisco, San Francisco, California, USA.; 2 Department of Chemical Engineering, Stanford University, Stanford, California, USA.; 3 Division of Pulmonary, Critical Care, Allergy and Sleep Medicine, UCSF, San Francisco, California, USA.; 4 Division of Pulmonary, Critical Care, and Sleep Medicine, University of Kansas School of Medicine, Kansas City, Kansas, USA.; 5 Department of Pathobiology, Lerner Research Institute, Cleveland Clinic, Cleveland, Ohio, USA.; 6 Division of Pulmonary and Critical Care Medicine, Department of Medicine, Brigham and Women’s Hospital, Boston, Massachusetts, USA.; 7 Division of Allergy, Pulmonary, and Critical Care Medicine, University of Wisconsin School of Medicine and Public Health, Madison, Wisconsin, USA.; 8 Department of Internal Medicine, Section of Pulmonary, Critical Care, Allergy and Immunologic Diseases, Wake Forest School of Medicine, Winston-Salem, North Carolina, USA.; 9 Department of Environmental and Occupational Health, University of Pittsburgh, Pittsburgh, Pennsylvania, USA.

**Keywords:** Cell biology, Pulmonology, Asthma, Molecular biology, Th2 response

## Abstract

Mucus plugs occlude airways to obstruct airflow in asthma. Studies in patients and in mouse models show that mucus plugs occur in the context of type 2 inflammation, and studies in human airway epithelial cells (HAECs) show that IL-13–activated cells generate pathologic mucus independently of immune cells. To determine how HAECs autonomously generate pathologic mucus, we used a magnetic microwire rheometer to characterize the viscoelastic properties of mucus secreted under varying conditions. We found that normal HAEC mucus exhibited viscoelastic liquid behavior and that mucus secreted by IL-13–activated HAECs exhibited solid-like behavior caused by mucin cross-linking. In addition, IL-13–activated HAECs shows increased peroxidase activity in apical secretions, and an overlaid thiolated polymer (thiomer) solution shows an increase in solid behavior that was prevented by peroxidase inhibition. Furthermore, gene expression for thyroid peroxidase (TPO), but not lactoperoxidase (LPO), was increased in IL-13–activated HAECs and both TPO and LPO catalyze the formation of oxidant acids that cross-link thiomer solutions. Finally, gene expression for TPO in airway epithelial brushings was increased in patients with asthma with high airway mucus plug scores. Together, our results show that IL-13–activated HAECs autonomously generated pathologic mucus via peroxidase-mediated cross-linking of mucin polymers.

## Abstract 2

IL-13 upregulates peroxidase activity in airway epithelial cells to autonomously generate pathologic mucus gels via disulfide cross-linking of mucin polymers.

## Graphical abstract

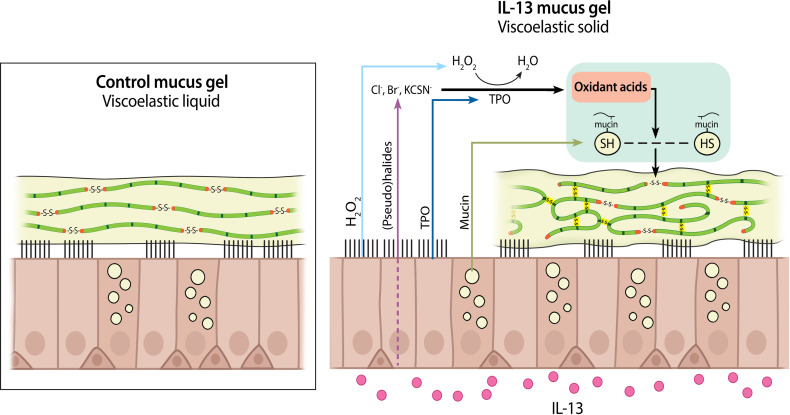

## Introduction

Airway mucus plugs are an important pathology in severe forms of asthma. For example, histopathologic analysis of lungs from fatal cases of asthma consistently show widespread airway mucus plugs that are a key cause of asphyxia and death in these patients (1, 2). In addition, image analysis of CT lung scans from patients with chronic severe asthma show prevalent and persistent mucus plugs and mucus plug scores that are strongly associated with measures of airflow obstruction and air trapping (3–5). There are no approved treatments for airway mucus plugs in severe asthma, and addressing this unmet clinical need requires a better understanding of the mechanism of formation of these plugs.

Rheological studies characterizing the biophysical properties of mucus plugs in patients with asthma show abnormally high elastic behavior (6), and characterization of inflammation in patients with asthma with mucus plugs shows upregulation of IL-13 in the airways (4). IL-13 is a plausible mediator of mucus plug formation in asthma because transgenic mice overexpressing IL-13 in their airways show mucus plugging (7), and IL-13–activated human airway epithelial cells (HAECs) form pathologic mucus gels with markedly reduced mucociliary movement (8). Taken together, these findings show that HAECs autonomously generate poorly transportable pathologic mucus when activated by IL-13, but the reasons why mucus becomes pathologic under IL-13–stimulated conditions are not known.

To investigate how IL-13 activates HAECs to cause pathologic mucus formation, it is first necessary to understand how the biophysical properties of the mucus change with IL-13. These biophysical properties are best measured using rheometry, but it has not been possible to apply controlled force to mucus gels secreted by HAECs when these cells are cultured under air liquid interface (ALI) conditions. We recently described how a magnetic microwire can be used to apply force to synthetic hydrogels in order to characterize their compliance and viscosity (9). We reasoned that this magnetic microwire technology is also well suited to characterize the mucus secreted by HAECs under ALI conditions, and we applied it here as a method of “live cell rheology” to compare the compliance and viscosity of mucus gels secreted under control and IL-13 conditions. In this way, we generated clues for the mechanisms by which IL-13 causes formation of solid mucus gels.

## Results

### Microwire movement in the mucus gel allows in situ quantification of mucus elasticity and viscosity but does not deform the epithelial cells.

**Figure 1:**
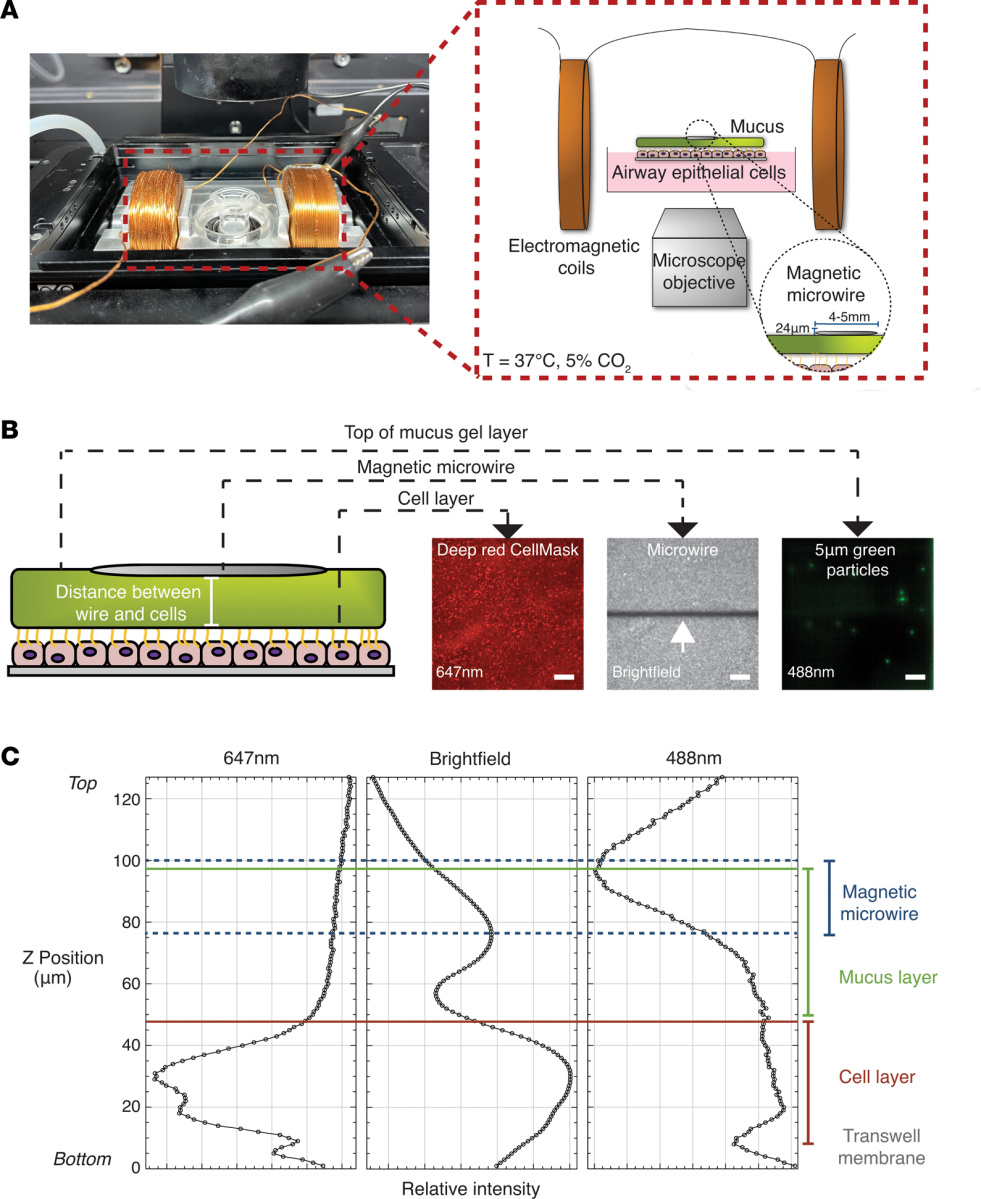
Design of the magnetic microwire rheometer and its application to measure the biophysical properties of mucus gels secreted by human airway epithelial cells grown at air-liquid interface culture. (**A**) Photograph and schematic of the magnetic microwire rheometer. (**B**) Illustration of the measurement of the distance between the microwire placed on top of the mucus gel and the surface of the epithelial cells. The wire position was identified using the bright-field channel of the microscope (white arrow), the cell layer was identified using the CellMask deep red at 647 nm, and the top of the mucus layer was estimated using fluorescent green particles imaged at 488 nm. Scale bar: 100 μm. (**C**) Plots of the relative intensity of the CellMask deep red signal (647nm), the bright-field signal, and the green particle signal (488 nm) to determine the position of the transwell insert membrane, the cell layer, the microwire, and the mucus layer.

HAECs cultured at ALI secrete an easily visible apical mucus gel that can be characterized with the magnetic microwire rheometer (MMWR) (Figure 1A). In initial experiments in mucus secreted by unstimulated HAECs (hereafter referred to as control mucus gels), we determined if the microwire placed on the surface of mucus contacts the cell surface. To do this, we measured the distance between the wire and the cell surface using the CellMask deep red plasma membrane stain (Invitrogen) to label the cell membrane, fluorescent particles (Duke Scientific) to label the mucus gel, and the microscope’s bright-field channel to identify the microwire (Figure 1, B and C). We found that the mean distance between the center of the wire and the epithelial cell surface was 57 μm, a distance included in calculations of the drag on the microwire. The cell layer was assumed to be a no-slip surface relative to the much softer and better-flowing mucus (10), and we observed no deformation of the cell surface due to wire motion on the mucus layer when magnetic force was applied to the wire (Supplemental Video 1; supplemental material available online with this article; https://doi.org/10.1172/jci.insight.181024DS1).

**Figure 2:**
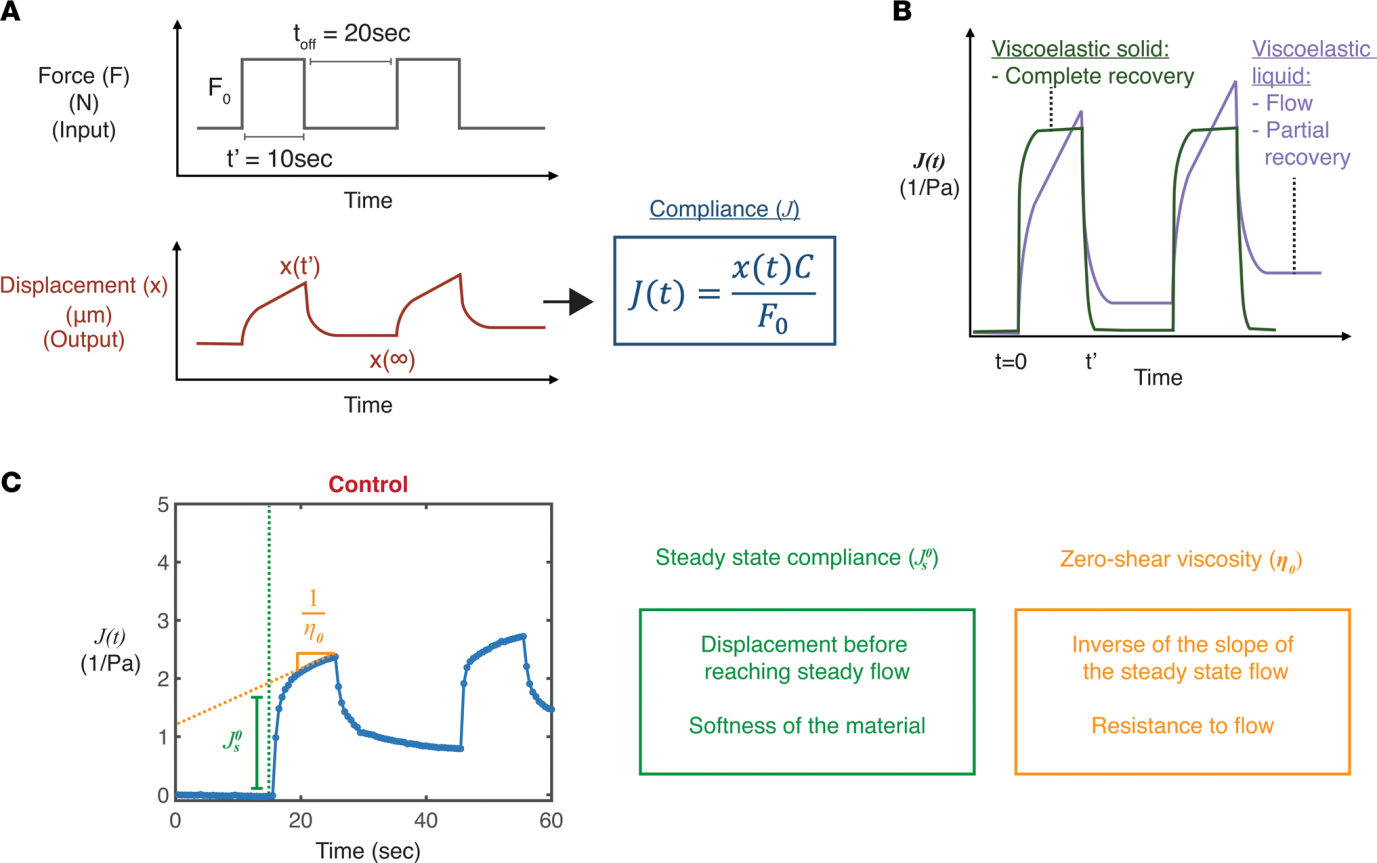
Creep compliance measurement reveals that unstimulated mucus shows viscoelastic liquid characteristics. (**A**) Schematic illustration showing how force, displacement, and time data can be used in a creep compliance test to calculate compliance over time. Compliance (*J*) is calculated as shown using equations in which and x(t) is the measured probe displacement, F_0_ is the amplitude of the applied force, and C is the geometric drag coefficient. (**B**) Illustration of example creep compliance curves for a viscoelastic liquid and a viscoelastic solid. (**C**) Representative evolution of the compliance over time for a control condition. The steady-state compliance (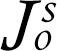), and the zero-shear viscosity (*n*_0_) are obtained by fitting (a) the compliance curve to determine the compliance value when the slope becomes linear and (b) the long-time slope, respectively. The steady-state compliance is a marker of the softness of the gel, while the zero-shear viscosity measures the resistance of the material to flow.

We next measured creep compliance of a control mucus gel using the MMWR. Creep compliance is defined by strain variations under constant unit stress. As illustrated in Figure 2A, a magnetic force was applied to the microwire probe in a step wave for 10 seconds followed by an “off” period of 20 seconds to allow the mucus gel to relax. Tracking the resulting microwire displacement and applied force values allows determination of the compliance of a material over time. Time-dependent creep compliance curves provide a visual representation of the elastic and viscous nature of the mucus. For example, as illustrated in Figure 2B, a viscoelastic solid will show complete recovery after force cessation while a viscoelastic liquid shows flow and only partial recovery, due to energy lost to viscous dissipation. Figure 2C provides an example of creep compliance measurements in a control mucus gel, and it reveals that the gel behaves like a viscoelastic liquid (Figure 2C). Specifically, with the magnetic force on, the microwire motion showed both elastic deformation and long-time flow. When the force was removed, there was a partial recoil, as the elastic portion was recovered, while the viscous energy had dissipated (Supplemental Video 2). By fitting the results from the creep compliance test with the viscoelastic Burgers model, the steady-state compliance and zero-shear viscosity of the mucus gel in situ on top of the epithelial cells could be determined (Figure 2C). Steady-state compliance (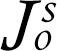) is the measure of the softness of a material and corresponds to the displacement in the compliance curve before reaching steady flow. Zero-shear viscosity (*n*_0_) accounts for the resistance to flow and is the inverse of the slope of the steady-state flow regime. The range of forces applied resulted in a consistent measurement of steady-state compliance and zero-shear viscosity (Supplemental Figure 1, A and B), indicating that the measurements were carried out in the limit of linear viscoelasticity.

### IL-13 activation of HAECs causes secretion of pathologic mucus that is liquefied by cleavage of disulfide bridges.

**Figure 3:**
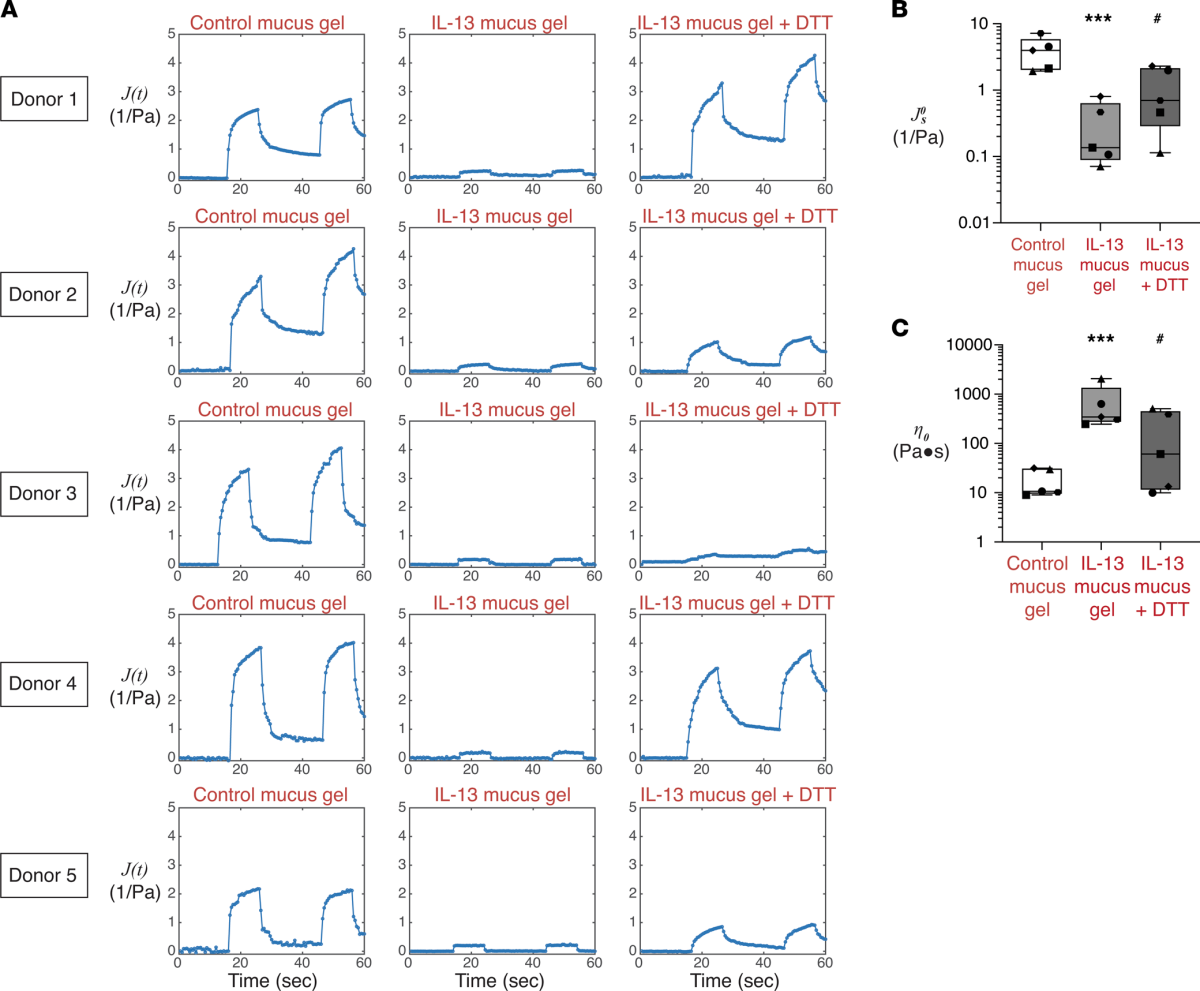
IL-13–activated airway epithelial cells secrete mucus gels with viscoelastic solid properties that are normalized by dithiothreitol. (**A**) Data from human airway epithelial cells from 5 donors illustrating creep compliance over time for mucus gels secreted under control conditions (control mucus gel), IL-13–activated conditions (IL-13 mucus gel), and dithiothreitol (DTT) treatment of IL-13 mucus gels (IL-13 mucus gel + DTT). (**B**) Summarized steady-state compliance data for creep compliance of control mucus gels, IL-13 mucus gels, and IL-13 mucus gels + DTT. (**C**) Summarized steady-state zero-shear viscosity for creep compliance of control mucus gels, IL-13 mucus gels, and IL-13 mucus gels + DTT. For **B** and **C**, the boxes show the interquartile ranges, while the error bars show the minimum and maximum values. Each symbol represent 1 donor (*n* = 5), and data are represent as the mean value of independent measurements from 2 or 3 transwell inserts of the same condition. *P* values were calculated using a ratio paired Student’s t test. Significantly different from control mucus gels, ***P < 0.001. Significantly different from IL-13 mucus gels, ^#^*P* < 0.05.

To confirm prior work of others (8, 11), we demonstrated that IL-13 activation of HAECs caused marked changes in gene and protein expression for gel-forming mucins (MUC5AC and MUC5B). Specifically, IL-13 activation resulted in a large increase in MUC5AC expression, whereas it caused a decrease in MUC5B expression (Supplemental Figure 2, B and C). To explore the effect of IL-13 on the creep compliance of HAEC mucus gels, we used the epithelial cell culture protocol illustrated in Supplemental Figure 2A. We found that, compared with control mucus gels, the creep compliance of mucus secreted by IL-13–stimulated HAECs (hereafter referred to as IL-13 mucus gels) was different. As above, the wire in control mucus gels displayed viscous flow when the magnetic force was applied and only partially recoiled after cessation of the force (Figure 3A and Supplemental Video 2). In contrast, under IL-13 conditions, the wire displayed decreased viscous flow when magnetic force was applied, and the wire recoiled nearly completely after cessation of the force (Figure 3A and Supplemental Video 3). Fitting the compliance versus time data to obtain the steady-state compliance (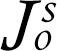) and zero-shear viscosity (*n*_0_) showed that the IL-13 mucus gels were consistently less compliant and more viscous than control mucus gels (Figure 3, B and C). Thus, IL-13 changed the mucus gel from a viscoelastic liquid to a viscoelastic solid. Therefore, IL-13 not only increased the concentration of MUC5AC in mucus, but it also caused an increase in mucin cross-link density. To determine if breaking mucin cross-links normalizes the biophysical behavior of IL-13 mucus gels, we exposed the IL-13 mucus gels to dithiothreitol (DTT), a chemical that breaks disulfide bridges. Under DTT conditions, the wire in IL-13 mucus gels displayed viscous flow when the magnetic force was applied and only partially recoiled after cessation of the force, mirroring wire behavior under control mucus conditions. Formal measures in multiple replicate experiments confirmed that DTT caused an increase in compliance of the IL-13 mucus gels and a decrease in viscosity (Figure 3, B and C). Taken together, these data demonstrate that IL-13 caused solid-like behavior in mucus gels by increasing mucin cross-links.

### IL-13 upregulates epithelial peroxidase activity in HAECs.

**Figure 4:**
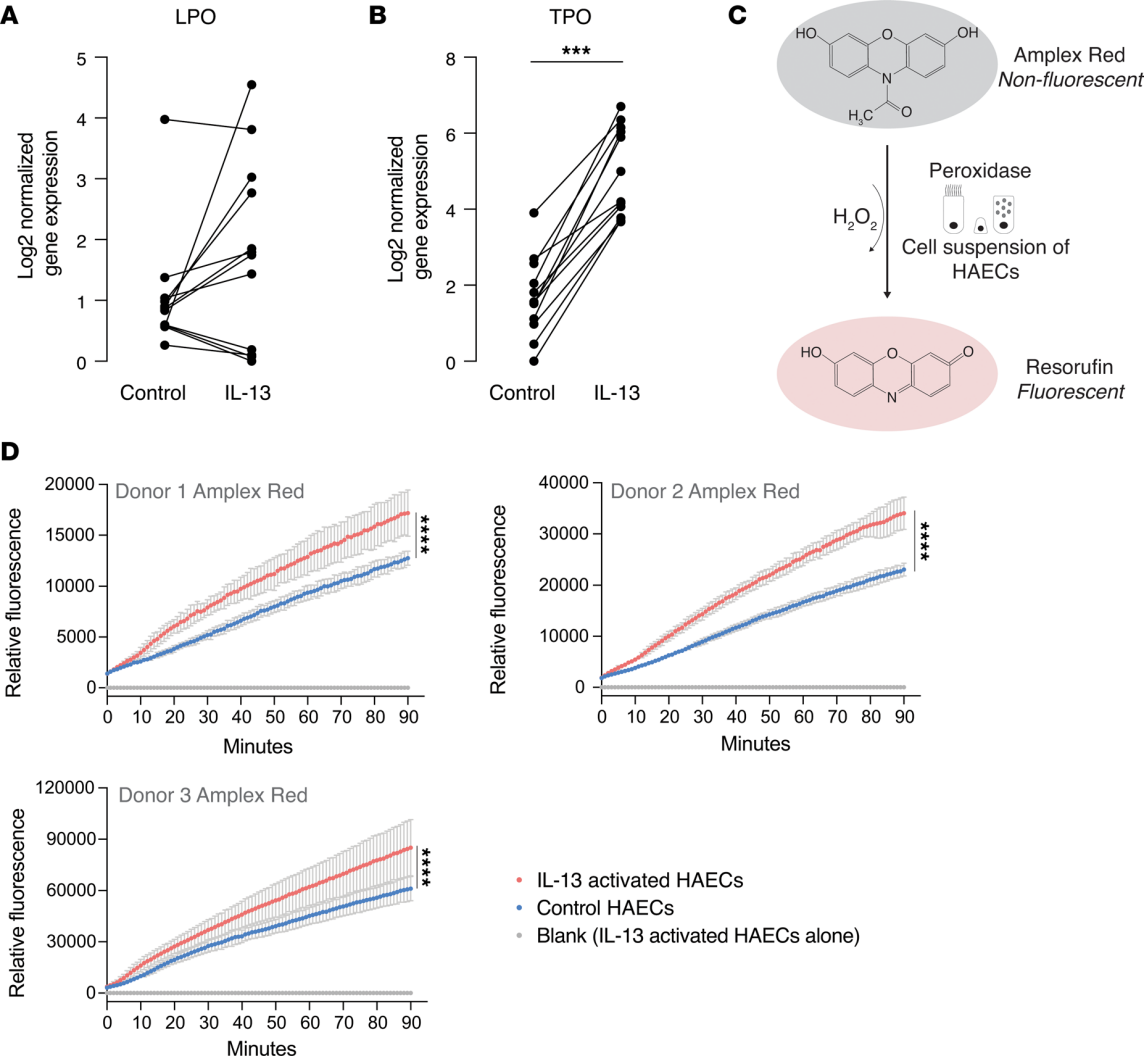
Epithelial peroxidase activity is increased in IL-13–stimulated HAECs. (**A**) IL-13 activation of human airway epithelial cells (HAECs) does not significantly change gene expression for lactoperoxidase (LPO) (*n* = 12 donors). (**B**) IL-13 activation of HAECs significantly increases gene expression for thyroid peroxidase (TPO) (*n* = 12 donors). *P* value was calculated using a 2-tailed paired Student’s *t* test. Significantly different from control, ***P < 0.001. (**C**) Schematic representation of the Amplex Red assay for peroxidase activity illustrating how the assay was done in 5,000 epithelial cells detached from transwell inserts by trypsin. (**D**) Peroxidase activity increases in IL-13–activated HAECs. The blank condition, corresponding to IL-13–stimulated cells alone, without Amplex red, shows that the cells are not autofluorescent at the assay wavelength. Experiments were performed in triplicate, and data are shown as mean ± SD. *P* values were calculated using a 1-way ANOVA test. Significantly different from control, ****P < 0.0001.

**Figure 5:**
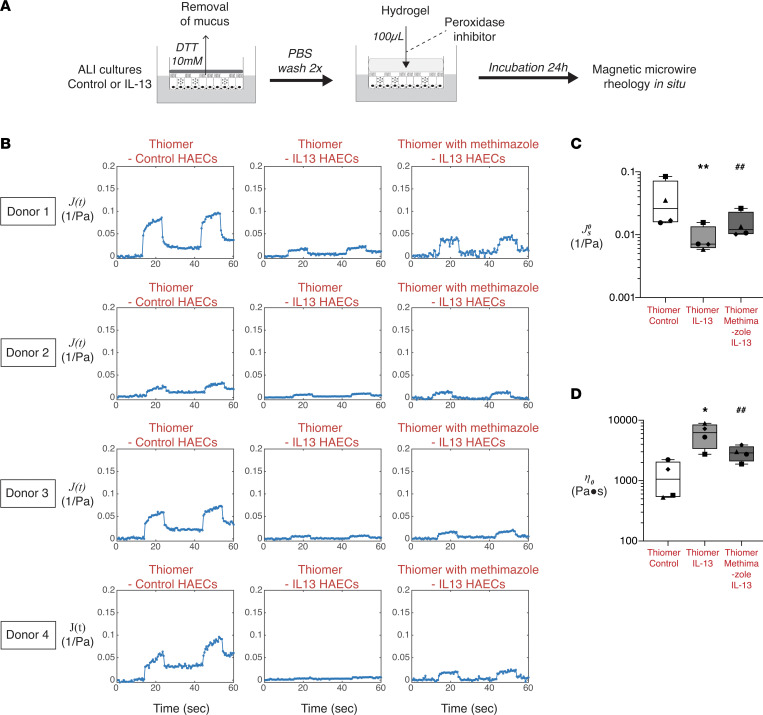
Cross-linking of a thiomer solution placed on the apical surface of IL-13–activated HAECs is prevented by inhibiting peroxidase activity. (**A**) Schematic illustrating how the thiomer solution was overlaid on the apical side of human airway epithelial cells (HAECs) at air-liquid interface (ALI) culture under control and IL-13 activation conditions. A methimazole condition tested the effect of peroxidase inhibition on thiomer cross-linking in IL-13–activated HAECs. (**B**) Data from 4 donors showing creep compliance over time of thiomer solutions overlaid on the apical surface of HAECs in control conditions, IL-13 conditions, and methimazole conditions. (**C**) Summarized steady-state creep compliance of thiomer solutions overlaid on HAECs in control conditions, IL-13 conditions, and methimazole conditions. (**D**) Summarized zero-shear viscosity of thiomer solutions overlaid on HAECs in control conditions, IL-13 conditions, and methimazole conditions. For **C** and **D**, the boxes show the interquartile ranges, while the error bars show the minimum and maximum values. Each symbol represents 1 donor (*n* = 4), and data are represented as the mean value of independent measurements from 2 or 3 transwell inserts of the same condition. *P* values were calculated using a ratio paired Student’s *t* test. Significantly different from control conditions, *P < 0.05; significantly different from control conditions, **P < 0.01; significantly different from IL-13 conditions, ^##^*P* < 0.01.

In considering the mechanism of IL-13–mediated mucin cross-links, we reasoned that epithelial cell peroxidases may play a role via the production of oxidants acids. Airway epithelial cells express lactoperoxidase (LPO) and thyroid peroxidase (TPO) (12, 13). LPO catalyzes the oxidation of thiocyanate into hypothiocyanous acid and plays a role in airway defense against bacterial infections (13). TPO is named for its high expression in thyrocytes where thyroglobulin iodination depends on the interaction of iodide, thyroglobulin, hydrogen peroxide, and TPO at the apical plasma membrane (14). TPO is membrane bound in this thyroglobulin iodination context in order to keep the iodination reaction in the storage follicle out of the cytoplasm. We confirmed that HAECs expressed both LPO and TPO but only TPO gene expression levels were increased after IL-13 stimulation (Figure 4, A and B). To determine if IL-13 activation increases peroxidase activity at the surface of HAECs, we incubated the cells with the Amplex red reagent, which produces resorufin as a fluorescent reporter of peroxidase activity (Figure 4C). We found that resorufin levels in the presence of IL-13–activated HAECs were significantly higher than in the presence of control HAECs (Figure 4D). To determine if this increase in peroxidase activity in epithelial cells can mediate the abnormal viscoelastic solid behavior of IL-13 mucus gels, we developed a thiomer-based assay illustrated in Figure 5A. In this assay, mucus was removed from the surface of the cultured epithelial cells, and a thiomer solution was layered on the apical surface of the cells for 24 hours. Compared with thiomer solutions overlaid on control epithelial cells, the thiomer gel overlaid on the IL-13 activated cells had lower steady-state compliance (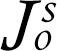) and higher zero-shear viscosity (*n*_0_) (Figure 5, B–D), indicating viscoelastic solid behavior. Notably, addition of a peroxidase inhibitor (methimazole) to the thiomer hydrogel solution blunted the IL-13–induced changes in steady-state compliance (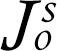) and zero-shear viscosity (*n*_0_) (Figure 5, B–D). These results show that epithelial cell peroxidase activity mediated the ability of IL-13 to generate pathologic mucus gels with solid-like behavior.

### LPO and TPO catalyze the formation of oxidant acids that promote covalent disulfide cross-links in thiomers.

**Figure 6:**
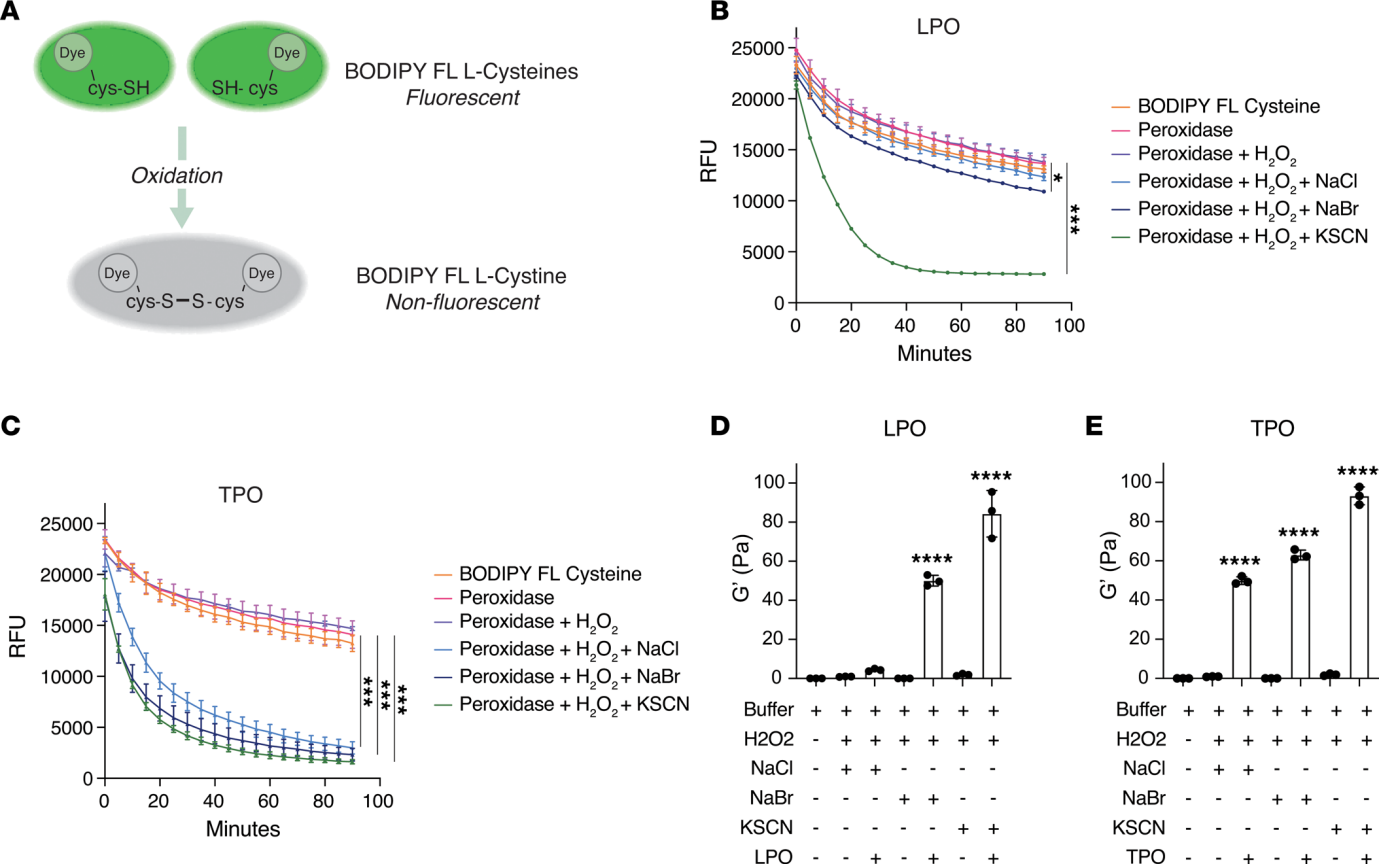
Epithelial peroxidases catalyze the production of oxidant acids that cross-link free cysteines and stiffen a thiomer solution. (**A**) Schematic representation of the BODIPY-labeled cysteine-cystine reagent assay. (**B**) Oxidant acid production measured by the quenching of the BODIPY-labeled cysteine reagent fluorescence in presence of lactoperoxidase (LPO), H_2_O_2_, NaCl, NaBr, or KSCN. Experiments were performed in triplicate. Data are shown as mean ± SD. *P* values were calculated using a 1-way ANOVA test. Significantly different from control, *P < 0.05; significantly different from control, ***P < 0.001. RFU, Relative fluorescent units. (**C**) Oxidant acid production measured by the quenching of the BODIPY-labeled cysteine reagent fluorescence in presence of thyroid peroxidase (TPO), H_2_O_2_, NaCl, NaBr, or KSCN. Experiments were performed in triplicate. Data are shown as mean ± SD. *P* values were calculated using a 1-way ANOVA test. Significantly different from control, ***P < 0.001. (**D**) Cross-linking of the thiomer solution, as revealed by changes in the elastic modulus (G’) of the gel after 1 hour incubation with LPO, H_2_O_2_, NaCl, NaBr, or KSCN. Experiments were performed in triplicate. Data are shown as mean ± SD. *P* values were calculated using a 1-way ANOVA test. Significantly different from control, ****P < 0.0001. (**E**) Cross-linking of the thiomer solution, as revealed by changes in the G’ of the gel after 1 hour incubation with TPO, H_2_O_2_, NaCl, NaBr, or KSCN. Experiments were performed in triplicate. Data are shown as mean ± SD. *P* values were calculated using a 1-way ANOVA test. Significantly different from control, ****P < 0.0001.

The oxidant acids generated by TPO and LPO activity are poorly characterized. In previous work, we have characterized the oxidant acids generated by eosinophil peroxidase (EPX), and we have used cone and plate rheometry to show that EPX-generated oxidant acids increase the elastic behavior of a thiomer gel (4). Here, we explored if LPO or TPO can act like EPX to generate oxidant acids that change the rheological behavior of a thiomer solution. To first characterize the oxidant acids that are generated by LPO and TPO, we used the BODIPY-labeled cysteine reagent, which fluoresces when the labeled cysteine is in its monomeric form and does not when it is in its oxidized (cystine) form (4) (Figure 6A). We found that, in the presence of H_2_O_2_ and halide ions, LPO and TPO both catalyzed production of oxidant acids that quench the fluorescence of the labeled cysteine reagent (Figure 6, B and C). Whereas LPO had high affinity for thiocyanate, low affinity for bromide, and no affinity for chloride (Figure 6B), TPO had high affinity for bromide, chloride, and thiocyanate (Figure 6C). In addition, to determine if LPO- or TPO-generated oxidant acids increase the elastic behavior of a thiomer gel (4), we again used cone and plate rheometry to measure the elastic modulus (G’) of the thiomer solution in the presence of H_2_O_2_ and halides. We found that LPO and TPO increased the G’ of the thiomer gel solution, indicating stiffening of the gel by disulfide cross-links in response to hypobromous or hypothiocyanous acids (Figure 6, D and E). As observed in the BODIPY-labeled cysteine assay and in contrast to TPO, LPO did not increase the G’ of the thiomer solution in presence of chloride (Figure 6, D and E). Thus, like EPX, both LPO and TPO can catalyze the formation of oxidant acids that promote covalent disulfide cross-links that modify the biophysical properties of thiomers.

### Airway epithelial cell expression of TPO is increased in patients with asthma who have airway mucus plugging.

**Figure 7:**
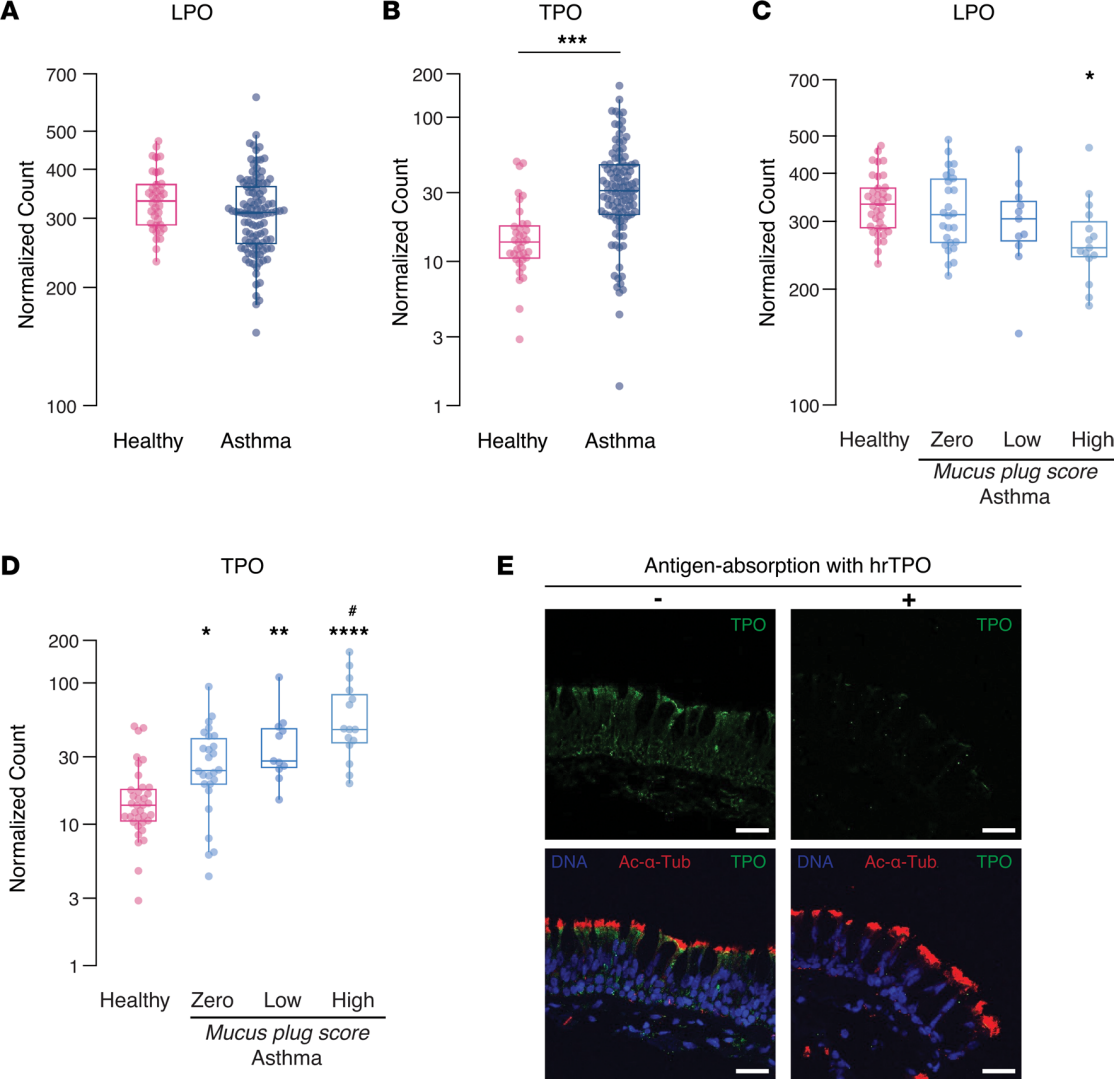
TPO gene expression is higher in patients with asthma, especially in patients with high airway mucus plug scores. (**A**) Gene expression for lactoperoxidase (LPO) in airway epithelial brushings is similar in patients with asthma (*n* = 104) and healthy controls (*n* = 36). (**B**) Gene expression for thyroid peroxidase (TPO) in airway epithelial brushings is higher in patients with asthma than in healthy controls. Significantly different than controls, ***P < 0.001. (**C**) LPO gene expression does not vary greatly among patients with asthma based on airway mucus plug scores determined from quantitative analysis of their CT lung scans. *P* values were calculated using a Kruskal-Wallis test. Significantly different from controls, *P < 0.05. (**D**) TPO gene expression is highest in patients with asthma with high airway mucus plug scores. *P* values were calculated using a Kruskal-Wallis test. Significantly different from controls, *P < 0.05; significantly different from controls, **P < 0.01; significantly different from controls, ****P < 0.0001; significantly different from patients with asthma with a mucus plug score of zero, ^#^*P* < 0.05. (**E**) Representative TPO immunostaining image of a tissue section from an endobronchial biopsy from a patient with asthma (*n* = 3 patients with asthma). Also shown is immunostaining for acetylated α-tubulin (Ac-α-Tub), which marks cilia and staining for DNA (DAPI) marking cell nuclei. The TPO immunostaining is most prominent at the apical membrane of ciliated epithelial cells. A control section shows how antigen preabsorption using human recombinant TPO (hrTPO) decrease the TPO signal intensity, providing evidence for the specificity of the TPO immunostaining signal. Scale bars: 30 μm.

To begin to explore the clinical implications of epithelial cell peroxidase activity for airway mucus plug pathology in asthma, we examined gene expression for LPO and TPO in epithelial brushings from patients with asthma in the Severe Asthma Research Program-3 (SARP-3) (Supplemental Table 2). The SARP-3 cohort is a longitudinal cohort study enriched in patients with severe asthma (15), and its protocol includes collection of airway epithelial brushings (during research bronchoscopy) and CT lung scans. The gene expression in the epithelial brushings has been previously measured using bulk RNA-Seq, and the CT lung scans have been previously analyzed for mucus plug scores (4, 16). When we used the RNA-Seq database to determine the expression of LPO and TPO in asthma, we found that TPO expression was significantly higher in patients with asthma than in healthy individuals, whereas expression of LPO was not (Figure 7, A and B). Additionally, when we used the mucus plug score data from the CT lung scans, we found no increase in epithelial cell LPO gene expression in asthma patient subgroups with high mucus plug scores (Figure 7C). In contrast, we found that the increase in epithelial cell TPO gene expression in asthma was driven by increases in expression in patient subgroups with high mucus plugs scores (Figure 7D).

Prior studies show that TPO is expressed in both the cytoplasm and at the apical plasma membrane of thyrocytes (14). Synthesis of thyroid hormone by thyrocytes depends on the interaction of iodide, thyroglobulin, hydrogen peroxide, and TPO at the apical membrane. We explored here if TPO also localizes to the apical membrane of airway epithelial cells in endobronchial tissue sections from patients with asthma available from the UCSF Airway Tissue Bank. Using tissue sections from 3 patients with asthma from this biobank, we applied immunofluorescence to show immunostaining for TPO in the apical region of ciliated cells in regions that were also positive for a cilia marker (acetylated α-tubulin). A representative image is shown in Figure 7E. Antigen-absorption control using recombinant human TPO showed reduced signals, confirming the specificity of the TPO immunostaining in this location (Figure 7E). Taken together, these data provide support for the hypothesis that IL-13–driven increases in TPO in airway epithelial cells could explain, at least in part, the mechanism of formation of mucus plugs in severe forms of asthma.

## Discussion

The formation of mucus plugs that occlude airways is a common pathology in asthma, but the mechanisms are not well understood. Here we used a MMWR to apply controlled force to epithelial cell mucus gels in situ to show that IL-13 changes the mucus gel to a viscoelastic solid that is liquefied by cleaving mucin disulfide cross-links. IL-13 also increases epithelial cell peroxidase activity, which catalyzes formation of oxidant acids to increase the disulfide cross-link density and decrease the compliance of thiomer hydrogels. TPO and LPO are expressed by airway epithelial cells, and we found that TPO gene expression is increased in airway epithelial cells from patients with asthma, especially in those with airway mucus plugging. Taken together, these data support a mechanism of pathologic mucus formation in asthma in which airway epithelial cells activated by IL-13 can autonomously generate pathologic mucus via peroxidase-mediated cross-linking of mucin polymers.

The MMWR is established as a rheological device ideal for characterizing small volumes of biomaterials (9), but it has not previously been validated for use in mucus gels secreted by airway epithelial cells. Here we show that the microwire used in the MMWR sits at the surface of the secreted mucus gel layer with no deformation or disturbance to the cell layer from the probe motion. Stress sweep experiments indicated that measures of creep compliance and viscosity were made in the linear viscoelastic regime. Similar rheological devices such as magnetic tweezers have used creep compliance measurements to characterize a range of biomaterials, including cytoplasm (17–19), filamentous actin (20, 21), and the cell cytoskeleton (22, 23), with the use of magnetic micron-scale particle probes. These methods require large custom magnet configurations to achieve the forces necessary to characterize viscoelastic materials, but the use of a magnetic microwire probe, which has much higher iron content than a magnetic bead, allowed consolidation of electromagnets into a highly portable device that can be mounted on virtually any microscope. An additional advantage of a microwire probe over magnetic beads includes the ability to average over an entire mucus layer secreted by cells in culture in 12 mm transwell inserts with a single probe and the ease of placement and removal of the probe with no need for mixing and minimal disturbance to the mucus gel.

In the experiments presented here, we used the MMWR to explore how IL-13, a key cytokine mediator of HAEC dysfunction in asthma (24, 25), mediates the formation of pathologic mucus gels. Prior work by others has shown that, when HAECs are activated by IL-13 under ALI culture conditions, mucus is formed that is abnormally adherent to the cell surface and poorly transportable by mucociliary motion (8). IL-13–mediated hypersecretion of mucins does not solely explain this abnormal behavior, so we deployed the MMWR to search for mechanisms. We discovered that IL-13 mucus is less compliant and more viscous than control mucus, indicating that IL-13 changes mucus from a viscoelastic liquid to a viscoelastic solid. We consider that the IL-13–induced transition of the mucus gel from liquid to solid state is likely to decrease mucus transport. The shearing motion of the cilia normally supplies a shearing stress to the “underbelly” of the mucus, which must overcome the resistance of the mucus in order for the mucus to flow. The cilia-mucus interface of a viscoelastic liquid mucus presents a “no-slip” boundary condition and will move with the cilia motion. The interface of a viscoelastic solid, on the other hand, is more likely to “slip” and remain motionless. A simple analogy would be sliding a knife through stiff butter. As the knife passes through the butter, it will shear the butter but only locally. The bulk of the butter will not be sheared. Sending a knife through mayonnaise (a viscoelastic liquid) will, on the other hand, shear the bulk of that material. Other factors might be responsible for the poorly transportable mucus such as MUC5AC tethering to the epithelium or MUC5AC’s more adhesive properties (8, 26), and mechanisms of mucus transport may also differ between large and small airways.

Our studies uncovered mechanisms by which IL-13 generates pathologic mucus gels in the airway. First, we found that DTT, which cleaves disulfide bridges between mucins, normalizes the compliance and viscosity of IL-13 mucus. This finding reveals the importance of mucin cross-links as a qualitative change in mucins that explain the low compliance/high elasticity of mucus under IL-13 conditions. Second, we found that IL-13–upregulated peroxidase activity in epithelial cells generated oxidant acids that cross-linked and solidified a thiomer solution. This finding reveals how epithelial cells can autonomously generate the oxidant machinery to drive disulfide cross-linking. Relevant here is the effect of IL-13 to increase MUC5AC gene expression relative to MUC5B (27). MUC5AC has more cysteine domains than MUC5B (28), and that may mean that MUC5AC is more susceptible to oxidant acid–induced cross linking.

Prior studies have shown that IL-13 regulates TPO expression in airway epithelial cells and that patients with asthma have high TPO expression in their bronchial epithelial brushings (12). We confirmed here that IL-13 upregulates TPO in airway epithelial cells, and we show that IL-13 did not significantly change LPO gene expression in these cells. We further confirmed that patients with asthma had increased TPO expression in their bronchial epithelial brushings. Together, these data show that, among the 2 epithelial cell peroxidases, TPO may be particularly important in peroxidase-mediated pathological mucus formation in asthma. Indeed, because the bronchial brushing data we analyzed had been collected from patients with asthma who had lung image–based mucus plug scores previously measured (4), we could link TPO gene expression to airway mucus plug scores in the same patients. In this way, we uncovered that increased TPO gene expression in the airway epithelium in asthma was driven by high TPO expression in patients with airway mucus plugging. Furthermore, our TPO immunostaining data in endobronchial biopsy tissue from patients with asthma show that TPO protein expression localized to the apical cell surface of ciliated epithelial cells. This TPO immunolocalization provided a mechanism for oxidant acids to be generated locally at the epithelial cell surface in close proximity to the necessary halides transported by ion channels and hydrogen peroxide produced by dual oxidases, which can also be regulated by IL-13 (12, 29). Oxidant acids generated close to the epithelial cell surface would increase the cross-link density of secreted mucins shortly after their secretion.

In summary, we successfully applied a MMWR to measure the rheology in situ of mucus gels secreted by IL-13–activated airway epithelial cells. In this way, we discovered that IL-13 mucus gels have low compliance/high elasticity that was caused by disulfide cross-linking of mucin polymers and mediated by epithelial cell peroxidase activity. Because IL-13 also increases expression of MUC5AC (27) and halide transporters (e.g., pendrin) (30) in airway epithelial cells, the pleotropic effects of IL-13, inducing qualitative and quantitative changes to the mucus, allow this cytokine to orchestrate a specific molecular environment in which occlusive mucus plugs form.

## Methods

### Sex as biological variable

Males and females were both included in the study.

### Patient biospecimens and data

We used patient data from several sources, including human biospecimens and data derived from lungs donated for transplantation, from the SARP-3 and from the UCSF Airway Tissue Bank. Further information is provided in Supplemental Methods.

### HAECs

HAECs were collected from the trachea of deceased organ donors via the California Transplant Donor Network following established procedures (31). HAECs from multiple different donors were expanded and cultured at ALI using the protocol published by Everman et al. (32), and live cell rheology experiments on secreted mucus were performed 21 days after air lifting (detailed methods are provided in Supplemental Methods).

### Thiolated hydrogel

Thiolated polymers (also referred to as thiomers) are useful as reagents to model the cross-linking behavior of cysteine-rich mucin polymers (4, 33). Here we overlaid a 1% solution of thiolated hyaluronic acid/phosphate buffer pH 7.4 (Glycosil, Advanced Biomatrix) on the apical surface of HAECs that were stripped of their native mucus gel in order to determine if epithelial cell products can modify the cross-linking behavior of the thiomer.

### MMWR

Rheometry measures deformation or stress of a material in response to an applied force or strain, respectively, but typical bulk rheological methods require large sample volumes and may result in sample destruction. We recently described how a MMWR can be used to characterize the rheology of a thiomer solution (9). As shown in Figure 1A, the MMWR instrument consists of 2 principal components: (a) 2 electromagnetic coils in anti-Helmholtz configuration; (b) a magnetic microwire probe (gift from Manuel Vazquez Villabeitia, Institute of Material Science of Madrid, Madrid, Spain) placed on the sample surface in the center of the 2 coils. The microwire position within the mucus layer is a balance of the upward force due to surface tension along the wire perimeter and the downward body force of gravity. The magnetic microwires are composed of Fe_76_Si_11_B_13_ alloy surrounded by a glass Pyrex coating and were magnetized along the longitudinal axis. The mean ± SD diameter of the microwires used in the experiments described here was 24.6 ± 1.2 μm (34). The length of the microwires was 5.3 ± 0.3 mm. During the in situ rheology experiments, the device was mounted on a confocal microscope to image the microwire displacement in response to an applied magnetic force. The magnetic microwire probe was aligned along its longitudinal axis parallel and centered to the axes of the electromagnetic coils. Current was fed to the coils by a power amplifier and controlled by a function generator, which produced a uniform magnetic field gradient between the coils and, as a result, a constant magnetic force on the microwire probe along its axis. The relationship between the current amplitude and applied magnetic force was linear, as shown in the force-current calibration curve previously established for this device and the magnetic microwires used in this work (9).

### Live cell rheology

#### Inhibition of cilia beating.

At 20 minutes before each live cell rheology experiment, 866 μL of cinnamaldehyde (30 mM, Sigma-Aldrich) was added to the basal chamber of the transwell insert to temporarily inhibit cilia beating.

#### Microwire experiments.

The transwell insert was transferred to an optical cell dish with 100 μL media to keep the cell membrane hydrated during the experiment. In total, 30 μL of PBS with or without DTT 10 mM (Bio-Rad) was added to the apical chamber right before microwire deposition using plastic tweezers. To prevent evaporation of the sample during the experiment, 100 μL of perfluorocarbon (Thermo Fisher Scientific) was added on the top of the mucus layer. The transwell insert and the optical dish were placed in the system such that the wire’s axis was aligned with the center of the coils. Images were acquired using a dry 20× objective on a Nikon SoRa spinning disk confocal inverted microscope in an environmental chamber (37°C, 5 %CO_2_) (Figure 1A). To make creep compliance measurements, a step force was applied to a microwire (5.3 mm length) for 10 seconds, followed by an “off” period of 20 seconds, twice over the course of 1 minute. The current amplitudes of 0.6 A and 2.5 A were applied to mucus gel of control and IL-13 conditions, corresponding to forces of 110 nN and 460 nN, respectively. For IL-13 mucus gels exposed to DTT, the amplitude was adjusted according to the donor’s response. When a thiomer solution was overlaid on the apical surface of HAECs, a current of 3.5 A was applied. Microwire motion was recorded by taking pictures every 500 ms for the duration of the experiment.

#### Creep compliance measurement.

The applied force, measured probe displacement, and geometric drag coefficient were used to calculate creep compliance over time (9) (Figure 2A). Following acquisition of the creep compliance data, the steady-state compliance and the zero-shear viscosity were extracted by fitting the data to the viscoelastic Burgers model (35) as previously described (9). The Burgers model is a general model of a viscoelastic material that incorporates both viscous flow and elastic deformation. This model is described by a combination of springs and dashpots, which represent elastic and viscous elements, respectively. The Burgers model is commonly used to describe the creep compliance behavior of viscoelastic materials. When a constant stress (force/area) is first applied to the material, there is an immediate elastic deformation (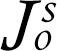) that is dampened, or slowed, by a viscous component. At long times, the material reaches a steady-state flow at a constant viscosity (*n*_0_). The creep compliance of a Burgers model material is described by the following equation:


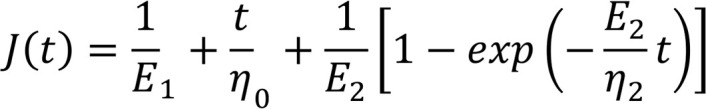


In this work, we fit the compliance versus time data with this model to determine the steady-state compliance 
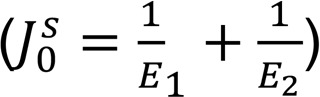
 and zero-shear viscosity (*n*_0_) of the sample; these terms describe the softness of the material and the resistance to flow, respectively. In our experiment, the compliance as a function of time, *J*(t), is defined as: 


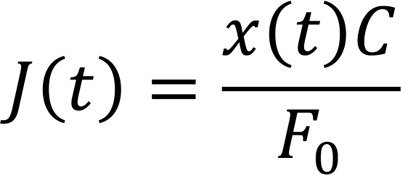
,

where x(t) is the measured microwire displacement, *F*_0_ is the magnitude of the applied magnetic force, and *C* is the geometric drag coefficient, which accounts for the shear stress applied to the sample from the applied force on the microwire. The data were fit relative to the onset of the applied force, such that the force and displacement were considered just as the force was turned on and until the force was turned off (t = 10 seconds).

For hydrodynamic purposes, the microwire was treated as a cylinder, and the cell layer was treated as a no-slip surface. The geometric resistance factor for a cylinder translating along the surface of a material with its longitudinal axis parallel to a no-slip surface is given by 


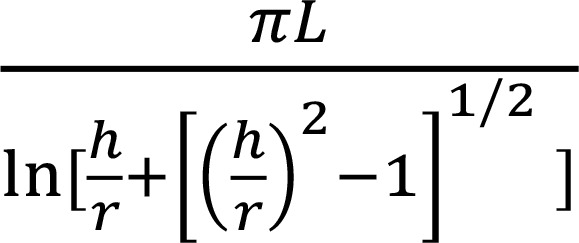
 (10, 36), where r and L are the radius and length of the microwire, respectively, and h is the height between the center of the microwire and the no-slip surface, or the thickness of the mucus layer in these experiments.

Additional details about membrane staining and mucus layer thickness measurement are provided in the online supplement.

### Cysteine cross-linking and peroxidase activity assays

A BODIPY-labeled cysteine reagent (Thermo Fisher Scientific) was used to characterize the halides and pseudohalides used by epithelial cell peroxidases to generate cross-linked cysteine, as previously described (4). The Amplex Red assay (Thermo Fisher Scientific) was used to quantify peroxidase activity of control and IL-13–activated HAECs. More details of both these assays are provided in Supplemental Methods.

### Gene and protein expression in HAECs

Detailed methods related to gene and protein expression measures in HAECs in the presence or absence of IL-13 activation are provided in Supplemental Methods.

### Statistics

Statistical analyses were performed using Prism 9 for PCR and rheology data. Differential gene expression analysis and data normalization of bulk RNA-Seq epithelial brushings were done with EdgR and DeSeq2 packages on R studio. The statistical tests employed to evaluate statistical significance are provided in figures legends, and *P* values lower than 0.05 are considered significant.

### Study approval

The SARP-3 and the UCSF Airway Tissue Bank studies have been reviewed and approved by the IRB at UCSF. All participant provided written consent.

### Data availability

The values corresponding to each data point in the different graphs can be found in the Supporting Data Values file. The RNA-Seq data for SARP are available at database of Genotypes and Phenotypes (dbGaP; https://www.ncbi.nlm.nih.gov/gap/) under accession no. phs001446. Investigators can contact the corresponding authors of the SARP studies at http://www.severeasthma.org/home.html to request access to the data through the ancillary study mechanism.

## Author contributions

MAL, MB, WWR, GGF, and JVF conceived the study. MAL, MB, and ARC conducted the experiments. MAL and MB analyzed the data. MC, SCE, EI, NNJ, BDL, WCM, and SEW performed bronchoscopies and collected bronchial brushings. PGW and SAC were involved in the sequencing of epithelial brush RNA. MT worked on SARP-3 demographic, clinical, and mucus plug score data. GGF and JVF supervised the study. MAL, MB, GGF, and JVF wrote the first draft of the manuscript, and all other authors reviewed and approved the final version of the manuscript. Co–first authorship order was determined according to the contribution to the study.

## Supplementary Material

Supplemental data

Supplemental video 1

Supplemental video 2

Supplemental video 3

Supporting data values

## Version 1 06/18/2024

In-Press Preview

## Version 2 08/08/2024

Electronic publication
