# Supplementary material for: Peroxidase-mediated mucin cross-linking drives pathologic mucus gel formation in IL-13–stimulated airway epithelial cells
Source: JCI Insight. 2024 Jun 18;9(15):e181024. doi: 10.1172/jci.insight.181024 (PMC11383604; doi:10.1172/jci.insight.181024)
Supplement: Supplemental data [file jciinsight-9-181024-s150.pdf]

Peroxidase-mediated mucin cross-linking drives pathologic mucus gel formation in IL-13-stimulated airway epithelial cells

Authors

Maude A. Liegeois[1§], Margaret Braunreuther[2§], Annabelle R. Charbit[1], Wilfred W. Raymond[1], Monica Tang[3], Prescott G. Woodruff[1,3], Stephanie A. Christenson[3], Mario Castro[4], Serpil C. Erzurum[5], Elliot Israel[6], Nizar N. Jarjour[7], Bruce D. Levy[6], Wendy C. Moore[8], Sally E. Wenzel[9], Gerald G. Fuller[2*] & John V. Fahy[1,3*]

Affiliations

[1] Cardiovascular Research Institute, University of California, San Francisco, San Francisco, CA, USA.

[2] Department of Chemical Engineering, Stanford University, Stanford, CA, USA.

[3] Division of Pulmonary, Critical Care, Allergy and Sleep Medicine, University of California, San Francisco, San Francisco, CA, USA.

[4] Division of Pulmonary, Critical Care, and Sleep Medicine, University of Kansas School of Medicine, Kansas City, Kansas.

[5] Department of Pathobiology, Lerner Research Institute, Cleveland Clinic, Cleveland, Ohio.

[6] Division of Pulmonary and Critical Care Medicine, Department of Medicine, Brigham and Women's Hospital, Boston, Massachusetts.

[7] Division of Allergy, Pulmonary, and Critical Care Medicine, University of Wisconsin School of Medicine and Public Health, Madison, Wisconsin.

[8] Department of Internal Medicine, Section of Pulmonary, Critical Care, Allergy and Immunologic Diseases, Wake Forest School of Medicine, Winston-Salem, NC, USA.

[9] Department of Environmental and Occupational Health, University of Pittsburgh, Pittsburgh, Pennsylvania.

[§] These authors contributed equally to this work

*** Corresponding authors**

John V. Fahy, M.D., M. Sc.,

Division of Pulmonary and Critical Care Medicine, Department of medicine,

University of California San Francisco,

Room 1303, Health Science East, 513 Parnassus Avenue

San Francisco, CA 94143

E-mail : John.Fahy@ucsf.edu

Gerald G. Fuller

Department of Chemical Engineering,

Stanford University,

Room 086, Shriram Center, 443 Via Ortega

Stanford, CA 94305

E-mail : ggf@stanford.edu

SUPPLEMENTAL MATERIAL AND METHODS

Human subject biospecimens and data

(i) Human Airway Epithelial Cells (HAECs)

Airway (tracheal) epithelial cells were harvested from the lungs of human organ donors whose lungs were deemed unsuitable for transplantation by the California Transplant Donor Network.

(ii) The Severe Asthma Research Program (SARP)-3

The Severe Asthma Research Program (SARP)-3 is a prospective multi-center longitudinal cohort study (1) in which 60 % of participants have severe asthma as defined by European Respiratory Society/American Thoracic Society (ERS/ATS) criteria (2). A subset of 104 asthma participants underwent research bronchoscopy during baseline characterization visits at a time of clinical stability. Some of these participants also underwent spirometry and optional computed tomography (CT) scan of the lungs at regular intervals (1). Mucus plug score was quantified as the sum of bronchopulmonary segments with mucus plugs on CT scan of the lungs inspected visually by thoracic radiologists (3). During bronchoscopy, epithelial cells were obtained by bronchial mucosal brushing and the cells were processed for RNA expression analysis by RNA bulk sequencing, using methods described in the RNA extraction and sequencing section. A cohort of 36 participants without asthma or other lung disease was recruited by SARP-3 centers and underwent bronchoscopy as healthy controls. The SARP-3 study protocol was approved by institutional review boards, and all asthma and healthy control participants signed informed consent. The clinical characteristics of the subjects are shown in Supplemental Table 2.

(iii) UCSF Airway Tissue Bank

We studied airway biopsies from 3 asthma patients stored in the Airway Tissue Bank (ATB) at the University of California, San Francisco (UCSF). The endobronchial biopsies had been collected in a prior allergen challenge research bronchoscopy study (4), although the biopsies studies here were from control conditions in which asthma patients had not been exposed to inhaled allergen. All three asthma patients had a history of asthma diagnosed by a physician, FEV1 as a percentage of predicted value >60% and methacholine hyperreactivity. None of the subjects was a smoker, used oral or inhaled steroids, or had history of respiratory tract infection in the last 6 weeks prior the bronchoscopy. The biopsy specimens were collected, formalin-fixed and paraffin embedded, as previously described (4).

Cell culture protocol for HAECs

Human airway epithelial cells (HAECs) were first expanded in rat tail collagen (Corning) pre-coated Petri dishes using medium 1 (47.5 % DMEM/F12 GlutaMAX, Gibco, 47.5 % F12 GlutaMAX, Gibco, 5 % heat inactivated FBS, Sigma-Aldrich, 24 µg/mL Adenine, Sigma-Aldrich, 10ng/mL human epidermal growth factor, Millipore Sigma, 400 ng/mL hydrocortisone, Sigma-Aldrich, 5 µg/mL insulin, Sigma-Aldrich, 8.6 ng/mL cholera toxin, Sigma-Aldrich) in the presence of antibiotics (50U/mL Pen-strep, 2.5 µg/mL amphotericin B and 50 µg/mL Gentamicin, Gibco) and ROCK inhibitor (10 µM, Selleckchem). The cells were harvested using Trypsin 0.25 % (Gibco) and then seeded onto 12 mm transwell inserts (Corning) coated with human placental collagen (Sigma) for rheology experiments or onto 6.5 mm transwell inserts (Corning) for PCR and immunofluorescence read-outs.

The cells were then cultured in medium 2 (50 % DMEM GlutaMAX, Gibco, 50 % BEBM, Lonza, BEBM supplements, Lonza, 50 µg/mL BSA, Sigma-Aldrich, 0.005 % Ethanolamine, Sigma-Aldrich, 0.3 µM $MgCl_2$, 0.4µM $MgSO_4$, 1 µM $CaCl_2$, Sigma-Aldrich, 30 ng/mL retinoic Acid, Sigma-Aldrich 10 ng/mL human growth factor, Gibco) until they reached confluence. Once the cells were confluent, they were air-lifted by removing media from the apical chamber and differentiated for 21 days using medium 3 (PneumaCult ALI complete basal medium, heparin 400 ng/mL, hydrocortisone 1 µM, Stemcell) with antibiotics (50 U/mL Pen-strep, 2.5 µg/mL amphotericin B and 50 µg/mL Gentamicin, Gibco). Starting from day 14 to day 21, the cells were stimulated with IL-13 10 ng/mL (Peprotech) every other day. As IL-13 stock solution contained BSA, BSA 0.05 % (dilution 1:1000, Sigma-Aldrich) was added in control condition media at the same frequency than IL-13 stimulation.

Characterizing gene and protein expression in HAECs in the presence and absence of IL-13 activation

Gene expression profiling: RNA was extracted using the Qiagen RNeasy mini kit, and the superscript VILO kit (Invitrogen) was used to synthetize cDNA. The expression of mucin genes, peroxidase genes and other HAEC genes activated by IL-13 was quantified using a 2 step RT-qPCR with the TaqMan Universal PCR Mix, using methods previously described (5) and primer and probe sequences shown in Supplemental Table 1. Housekeeping genes were GAPDH, PPIA and EEF1A1.

Immunostaining of air-liquid interface whole-mounts: Cell culture transwell inserts were fixed using Carnoy's solution for 30 minutes at room temperature. The inserts were then

washed 2 times in methanol and 2 times in ethanol and then stored in ethanol 100%. Before immunostaining, membranes were excised from the inserts and rehydrated through a graduated ethanol series and water. Antigen retrieval was performed at 99°C using a citrate buffer (Vector Laboratories) for 20 minutes. Membranes were cooled down for 1 hour before cell permeabilization in Triton X 0.1 % buffer (Sigma-Aldrich). Prior to primary antibody incubation, membranes were incubated in 10 % goat serum (Gibco) – 0.05% Tween 20 (Sigma-Aldrich) – PBS buffer for 2 hours to prevent unspecific binding. Membranes were stained overnight at 4 °C with a mouse monoclonal anti-MUC5AC antibody (1:300, clone 45M1, Invitrogen) and a rabbit polyclonal anti-MUC5B antibody (1:200, polyclonal, Sigma-Aldrich). The next day, secondary antibodies; Cy 3 goat anti-mouse and Cy5 goat anti-rabbit (Jackson Immunoresearch); were added at the concentration 1:300 for 2 hours. Nuclei were stained with DAPI (1:1000, Calbiochem). Images were acquired with a confocal laser-scanning microscope Olympus Fluoview 10i at 60x magnification.

Live cell plasma membrane staining

To visualize the cell layer during the live cell rheology experiments, a 1:1000 dilution of CellMask deep red plasma membrane stain (Invitrogen) was added to medium 3 and then added to the basal chamber of the cells. On the morning of the experiment, the cells were stained for 10 minutes at 37 °C in a humidified atmosphere contain 5 % CO2, and then the CellMask-containing media was removed. The basal chamber was washed with PBS, and the cells were put back into medium 3 until the start of the experiment.

Mucus layer thickness measurement

For hydrodynamic purposes, the microwire was approximated as a cylinder translating along its axial axis parallel to a no-slip surface. In this scenario, the resulting geometric drag coefficient is a function of the microwire diameter, length, and distance from the cell layer (6, 7). Microwire position relative to the cell layer was first determined by taking a z-stack of images from the bottom of the transwell to the top of the microwire on 647 nm, brightfield, and 488 nm channels (Figure 2C). Deep red CellMask staining was used to determine the boundaries of the cell layer and 5 µm fluorescent green polystyrene particles (Duke Scientific) were added to the mucus layer surface to verify the microwire position in the mucus layer. The size of the particles was chosen to ensure the particles were much larger than the mesh size of the mucus layer (~100-500 nm) (8, 9) and thus would stay at the surface of the mucus layer and not diffuse into it. The intensities of each channel were used to measure the relative positions of the cell surface, microwire, and particles. The z-position of bottom of microwire and center of the particles were determined by a local minimum and global maximum in intensity, respectively. Because CellMask deep red stains the cell membrane, the peak in intensity was associated with the center of the cell layer, so the intensity of the minimum between the membrane and cell layer maxima was taken as the indication for the boundaries of the cell layer (bottom and top). In all MMWR experiments, the mucus layer thickness was measured as the distance between the cell layer (stained with CellMask Deep Red) and the microwire (imaged with brightfield) measured from relative intensity of the z-stack images (described

in the online supplement). The thickness of the mucus layer did vary between samples, the average thickness was 57 μm with a standard deviation of 16 μm.

BODIPY-labeled cysteine cross-linking assay

BODIPY-labeled cysteine reagent was generated as previously described (3). Briefly, BODIPY FL L-Cystine (1 mM, Thermo Fisher Scientific) was incubated in TCEP gel (Thermo Fisher Scientific) for 30 minutes at room temperature to generate the BODIPY-labeled cysteine reagent. This reagent (8 μM) was diluted in phosphate buffer and incubated in presence of bovine lactoperoxidase (10n g/mL, Innovative Research Inc.) or thyroid peroxidase (1 ng/mL), NaBr (50 μM, Sigma- Aldrich), NaCl (50 μM, Sigma-Aldrich), KSCN (50 μM, Fisher Science Education) and H_2O_2 (2 μM, Sigma-Aldrich). The decrease in fluorescence (490 nm/520 nm) was monitored on a plate reader over 90 minutes at 37 °C (BioTek Instruments).

Thiomer cross-linking assays

The Glycosil thiol-modified Hyaluronan ("Thiomer", Advanced Biomatrix) was reconstituted to a 1 % concentration using the Buffer A provided by the company. To assess the thiomer cross-linking arising from oxidant acids derived from LPO and TPO acting as catalysts in the oxidation of (pseudo)halides by hydrogen peroxide, the following reagents were added to Buffer A (concentrations are given as final concentration): H_2O_2 (2 mM), NaCl (2 mM), NaBr (2 mM), KSCN (2 mM), LPO (1 ng/mL), or TPO (1 ng/mL). The elastic moduli (G') of the thiomer solutions were determined after 1 hour of incubation, using a cone and plate rheometer, which operated at 1Hz with a 5% strain (TA

instrument). To evaluate thiomer gel cross-linking through peroxidase activity on the apical side of epithelial cells, apical chambers of ALI-cultured cells in 12 mm transwell inserts were washed with 10 mM DTT followed by 2 PBS washes. Subsequently, 100 μL of the thiomer solution was added to each apical chamber. The plates were then incubated for 24h at 37 °C and 5 % CO_2 (Figure 5A). Following incubation, the thiomer gel solution biophysical properties were measured using the magnetic microwire rheometer using the same method described in the “Live cell rheology” section. In specific experimental conditions, methimazole (Sigma-Aldrich) was dissolved in the same buffer and added to the thiomer solution at a final concentration of 1 μg/mL.

Amplex Red assay

Briefly, the apical side of the cells was washed with 10 mM DTT to remove mucus. Cell suspensions were generated from air-liquid interface cultures by incubating the culture in 0.25 % Trypsin (Gibco) for 15 minutes. The cells were counted, and 5,000 cells of each condition were added to microplate well in 50 µL of PBS. Following the addition of 50 µL of the Amplex Red/H_2O_2 working solution, resorufin production was assessed by fluorescence measurement (560 nm/590 nm) on a plate reader over 90 minutes at 37 °C. Experiments for each donor were conducted in triplicate.

RNA extraction, library preparation, sequencing and analysis of epithelial brushings

Brush samples were taken from the airway epithelial lining of the lower lobe at points of segmental or subsegmental carina. After extraction with the Qiagen miRNeasy kit, RNA

concentration and quality were assessed using the ThermoFisher NanoDrop spectrophotometer and the Agilent 2100 Bioanalyzer, respectively. The libraries were generated using the Illumina TruSeq Stranded Total RNA with the Ribo-zero GOLD kit. Sequencing was performed on an Illumina HiSeq platform (UCSF Sandler Genomics core) using one hundred base-pair paired-end reads. FASTQ files underwent quality control and were mapped to the Ensembl GRCh38 human genome reference using the STAR alignment tool (10). Read counts from bulk RNA sequencing were analyzed in R. We utilized EdgeR to evaluate the differential expression levels of LPO and TPO between healthy individuals and those suffering from asthma (11). Subsequently, for data visualization and correlation analysis with the mucus plug score, we employed DESeq2 to normalize the gene counts for LPO and TPO (12). The resulting normalized gene counts and the corresponding mucus score data are available in Supplemental Table 3.

Immunostaining of bronchial biopsies from asthma patients

To confirm TPO localization at the membrane of epithelial cells in bronchial biopsies from the UCSF Airway Tissue Bank, 5 μm tissue sections were cut. They were then deparaffinized in xylene and rehydrated through a graduated ethanol series and water. Antigen retrieval was performed using the IHC Antigen Retrieval Solution (Invitrogen) for 30 minutes. Slides were cooled down for 1 hour before cell permeabilization in Triton X 0.1 % buffer (Sigma-Aldrich). To prevent unspecific binding, tissue sections were preblocked in 10% goat serum (Gibco) – 0.05 % Tween 20 (Sigma-Aldrich) – PBS buffer for 2 hours. Membranes were stained overnight at 4 °C with a mouse monoclonal anti-acetylated α-tubulin antibody (1:200, clone 6-11B-1, Santa Cruz Biotechnology) and a

rabbit monoclonal anti-TPO antibody (1:200, clone EPR5380, Abcam). For antigen-absorption experiments, the anti-TPO antibody was incubated overnight at 4 °C with human recombinant TPO (2 μg/mL) prior staining. Cy3 goat anti-mouse and Cy5 goat anti-rabbit (1:300, Jackson Immunoresearch) were used as secondary antibodies for 2 hours. Nuclei were stained with DAPI (1:1000, Calbiochem). Images were acquired with a confocal laser-scanning microscope Olympus Fluoview 10i at 60x magnification.

Supplemental Figures and Legends

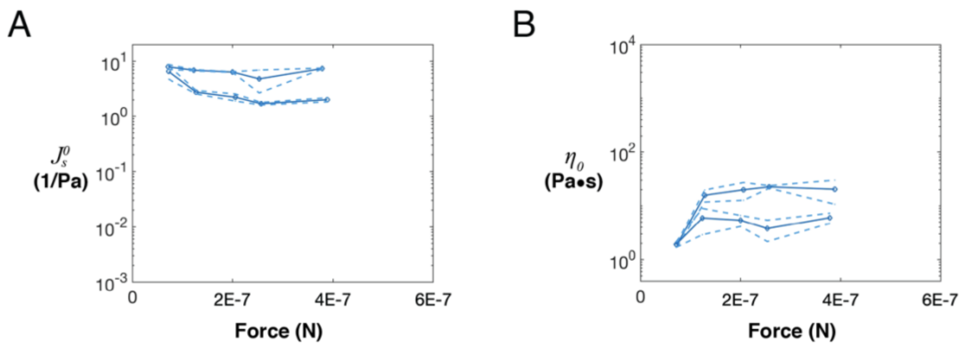

Supplemental Figure 1. A, Stress sweep showing the constant relationship between the applied force and the measured steady-state compliance. **B,** Stress sweep showing the constant relationship between the force applied and the measured zero-shear viscosity. For a wire of length 5.3 mm, this range of applied forces (100-400 nN) corresponds to currents of 0.5-2 A. The circle and diamond symbols indicate measurements associated with two independent transwell inserts from the same donor. The stress sweep is showed as the average of the measurements +/- the standard deviation.

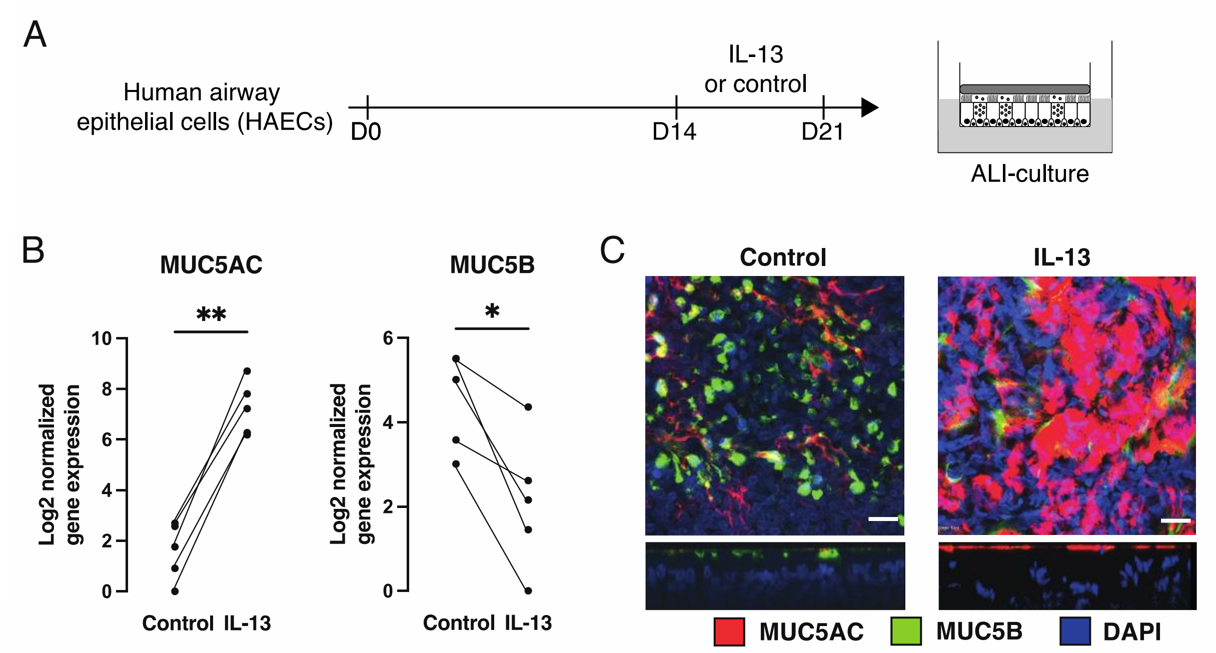

Supplemental Figure 2. A, Experimental pipeline of the human airway epithelial cells (HAECs) at air liquid-interface (ALI) cultures. **B,** IL-13 activation of HAECs significantly increases gene expression for MUC5AC and decreases MUC5B gene expression (n=5 donors). P values were calculated using a two-tailed paired Student's t test. *Indicates significantly different from controls, *P*<0.05. **Indicates significantly different from controls, *P*<0.01. **C,** Representative top and orthogonal views of whole mount pictures of control and IL-13-activated HAECs at ALI-cultures. The pictures show an increase in MUC5AC immunostaining and a decrease in MUC5B immunostaining after IL-13 activation. The membranes were also stained for DNA (DAPI), marking cell nuclei. Scale bar = 20 μm.

Supplemental Tables

Supplemental Table 1. Primer and probe sequences.

Gene		Preamplification primer sequences		Primer and probe sequences	Dye
**GAPDH**	Forward RT	CAATGACCCCTTCATTGACCTC	TaqMan Forward	GATTCCACCCATGGCAAATTC	
	Reverse RT	CTCGCTCCTGGAAGATGGTGAT	TaqMan Probe	CGTTCTCAGCCTTGACGGTGCCA	5-FAM/3-BHQ
			TaqMan Reverse	GGGATTTCCATTGATGACAAGC	
**PPIA**	Forward RT	ATGAGAACTTCATCCTAAAGCATACG	TaqMan Forward	ACGGGTCCTGGCATCTTGT	
	Reverse RT	TTGGCAGTGCAGATGAAAAACT	TaqMan Probe	ATGGCAAATGCTGGACCCAACACA	5-FAM/3-BHQ
			TaqMan Reverse	GCAGATGAAAAACTGGGAACCA	
**EEF1A1**	Forward RT	ATGACCCACCAATGGAAGCA	TaqMan Forward	CTGAACCATCCAGGCCAAAT	
	Reverse RT	TGTGAGCCGTGTGGCAAT	TaqMan Probe	AGCGCCGGCTATGCCCCTG	5-FAM/3-BHQ
			TaqMan Reverse	GCCGTGTGGCAATCCAAT	
**MUC5AC**	Forward RT	CTGCTTCTGCAACGTGGCT	TaqMan Forward	CACCATATACCGCCACAGAGAC	
	Reverse RT	TGGGCATCCCAGCTCAGT	TaqMan Probe	TCGCTGGCCATTGCTATTATGCCC	5-FAM/3-BHQ
			TaqMan Reverse	GTCAACCCCTCTGACCACTTG	
**MUC5B**	Forward RT	TTGAGGACCCCTGCTCCCT	TaqMan Forward	CGATCCCAACAGTGCCTTCT	

	Reverse RT	AGGCGTGCACATAGGAG GAC	TaqMan Probe	CAACCCCAAGCCCTTCC ACTCGA	5-FAM/3-BHQ
			TaqMan Reverse	CCTCGCTCCGCTCACAG T	
**LPO**	Forward RT	TGTCAACCAGGAGGTCTC AGACCATGGACTACCCT	TaqMan Assay	Hs00976400_m1	FAM-MGB
	Reverse RT	CGGGCTTCCTGGTAGAG CTTCTCTCCATCCCA			
**TPO**	Forward RT	TGCAAACATGTCTGGATG TCTCCCTTACATGCTGC	TaqMan Assay	Hs00892519_m1	FAM-MGB
	Reverse RT	GGGCAGTGGGAACCCGT TGTACAAGAAGCC			

Supplemental Table 2. Clinical and demographic features of SARP participants.

	**Healthy**	**Asthma**
**n**	36	104
**Age (years), mean (SD)**	42.0 (13.6)	41.5 (13.3)
**Female, n (%)**	21 (58.3)	72 (69.2)
**BMI, mean (SD)**	28.2 (5.6)	31.5 (8.9)
**Race, n (%)**		
**White**	25 (69.4)	65 (62.5)
**Black/African American**	7 (19.4)	30 (28.8)
**Asian**	3 (8.3)	1 (1.0)
**Other/Multiple**	1 (2.8)	8 (7.7)
**Hispanic, n (%)**	0 (0)	5 (4.8)
**Pre-BD FEV1 % predicted, mean (SD)**	101.42 (13.01)	77.91 (20.60)
**Pre-BD FVC % predicted, mean (SD)**	103.46 (14.26)	90.94 (19.27)

Supplemental Table 3. TPO and LPO normalized counts and mucus plug scores.

Status	LPO Normalized count	TPO Normalized count	Mucus plug score
Asthma	248.4129917	70.00161905	8.5
Asthma	387.8842733	7.949697563	0
Asthma	317.8225219	34.49884164	NA
Asthma	251.4863332	60.841662	NA
Asthma	334.3714283	17.34672345	NA
Asthma	309.4056212	40.76185625	NA
Asthma	235.6559365	25.80826223	NA
Asthma	361.1942379	29.69287897	0
Asthma	341.1868776	7.616174988	NA
Asthma	225.1713423	29.2688914	NA
Asthma	411.6239978	12.46618705	NA
Asthma	153.3523368	45.73181396	2
Asthma	368.5141864	21.21929736	NA
Asthma	325.908569	30.66264811	NA
Asthma	375.8033545	51.97017433	2.5
Asthma	265.3095382	94.03968761	0
Asthma	292.1592852	53.1947448	0
Asthma	319.8225856	31.26529894	NA
Asthma	460.887793	25.86571862	1.5
Asthma	190.2452155	165.4958396	12.5
Asthma	287.072839	46.79867593	4
Asthma	466.1422946	22.15778115	8.333333333
Asthma	313.0756106	12.95557462	NA
Asthma	308.633163	35.98200058	NA
Asthma	268.3121845	47.39318405	17
Asthma	330.6370393	39.08744615	7
Asthma	289.0590269	22.03425574	0
Asthma	274.4326419	14.91750747	0.5

Asthma	306.3860103	52.09523066	NA
Asthma	293.2847132	32.68107839	NA
Asthma	256.1044398	40.90482554	16
Asthma	181.0937127	108.1265561	12.5
Asthma	353.4976085	19.43047112	4
Asthma	317.9147714	42.15171209	0.5
Asthma	244.9520739	76.65622303	8
Asthma	305.4151073	22.55121724	NA
Asthma	229.7216569	103.9882586	NA
Asthma	316.168891	23.17736286	0
Asthma	304.5066839	27.34959316	0.5
Asthma	346.2189992	21.13994025	1
Asthma	307.3653755	15.0035874	NA
Asthma	251.8729795	22.30861466	0
Asthma	421.8003136	6.072755471	0
Asthma	289.07614	4.284605115	0
Asthma	264.0085057	19.21066313	0
Asthma	277.6237323	28.01583158	2
Asthma	240.6502692	46.7940278	14.5
Asthma	361.4788566	33.66078735	0
Asthma	310.2299062	42.23665403	0
Asthma	259.5770125	24.60018721	1.5
Asthma	205.4330008	132.614168	5.5
Asthma	276.9230082	45.76224696	NA
Asthma	255.205453	34.31981119	0
Asthma	231.3163598	19.25382923	0
Asthma	326.1128876	24.77448263	NA
Asthma	426.4599373	6.620114042	NA
Asthma	314.4144113	17.32991016	0
Asthma	310.9515344	61.37284987	NA

Asthma	185.2499411	82.94217802	NA
Asthma	261.8081591	56.2848879	NA
Asthma	317.1173162	20.81768874	NA
Asthma	369.6177324	9.084133311	NA
Asthma	308.0476167	7.847553982	NA
Asthma	250.6877716	89.69737946	NA
Asthma	447.3915535	20.42510111	NA
Asthma	313.1978406	30.39023476	NA
Asthma	610.2416143	1.364881722	NA
Asthma	231.0049155	28.70006946	NA
Asthma	367.8641508	24.25199251	NA
Asthma	403.0418447	58.00597782	0
Asthma	264.8055562	37.86733726	NA
Asthma	293.1396635	67.36138008	NA
Asthma	349.6768051	12.94590593	NA
Asthma	424.4824776	32.70120083	NA
Asthma	394.4515082	6.990140167	NA
Asthma	423.0076771	6.347857122	0
Asthma	455.6219902	35.50938386	0
Asthma	381.0947788	47.82784814	0
Asthma	327.0088532	12.8088655	0
Asthma	489.0441039	24.23999482	0
Asthma	251.0640797	88.66143543	4
Asthma	216.6021353	44.64989862	0
Asthma	272.1833799	31.59629047	0
Asthma	348.1495304	15.54252776	NA
Asthma	320.1533061	28.10237462	NA
Asthma	203.2618939	43.45802836	NA
Asthma	235.4945885	73.13469099	NA
Asthma	260.0939454	20.65112818	0

Asthma	234.1866501	42.01793275	0
Asthma	325.3827925	36.1035937	NA
Asthma	375.85208	30.48680529	NA
Asthma	243.345132	109.7160646	0.5
Asthma	274.1821765	36.28533063	10.5
Asthma	378.6164048	26.81900729	NA
Asthma	329.8660311	48.98535483	0.5
Asthma	395.2436009	23.72021182	0
Asthma	311.6852283	26.67445845	4
Asthma	250.4874108	68.51128089	NA
Asthma	242.6439244	27.03632049	NA
Asthma	312.340747	110.6067329	NA
Asthma	369.1719444	33.31373328	NA
Asthma	371.6127308	34.5817814	NA
Asthma	377.8802228	37.77212077	NA
Asthma	357.6424011	21.31504791	NA
Healthy	312.992567	11.42631353	NA
Healthy	354.3715106	11.3644762	NA
Healthy	276.5360232	10.27627582	NA
Healthy	291.0801219	10.35017362	NA
Healthy	395.5518895	7.682761628	NA
Healthy	310.7973328	15.83789098	NA
Healthy	323.8097298	28.61388955	NA
Healthy	431.1633058	22.168594	NA
Healthy	342.1374505	11.31131172	NA
Healthy	289.0389675	11.14512695	0
Healthy	305.4525878	12.10987009	0
Healthy	281.0176673	14.05976639	NA
Healthy	365.5467088	10.61464432	NA
Healthy	357.3343894	9.064025346	NA

Healthy	367.6703348	17.44632314	NA
Healthy	428.7806983	13.67786764	NA
Healthy	456.5963281	29.86636318	NA
Healthy	298.3237612	8.406825518	NA
Healthy	276.3428229	11.64516456	NA
Healthy	265.8211993	12.46120161	NA
Healthy	250.4322482	45.81738566	NA
Healthy	265.241299	26.9741299	NA
Healthy	282.1551934	14.04111507	NA
Healthy	395.3269731	18.209917	NA
Healthy	232.6399368	49.30214581	NA
Healthy	319.7157818	2.882207327	NA
Healthy	346.5568409	4.679430446	NA
Healthy	471.8522243	15.13177636	NA
Healthy	348.2922202	16.96926948	NA
Healthy	336.0235025	15.21594309	NA
Healthy	350.9324794	18.20606212	0
Healthy	328.9119051	48.01491392	0
Healthy	340.3449216	16.4302307	NA
Healthy	287.8709806	9.701533862	NA
Healthy	432.8101056	7.416961689	NA
Healthy	392.5743605	13.56914535	NA

Supplemental Videos and Legends

Supplemental Video 1. Representative video of the cell layer during creep compliance test on one control condition. (Left: Brightfield, right: CellMask membrane staining, 647 nm)

Supplemental Video 2. Representative video of the creep compliance test on one control condition. (current of 0.6 A). The dashed bar indicates the initial microwire position.

Supplemental Video 3. Representative video of the creep compliance test on one IL-13-stimulated condition. (current of 2.5 A). The dashed bar indicates the initial microwire position.

Supplemental references

1. Teague WG, et al. Baseline Features of the Severe Asthma Research Program (SARP III) Cohort: Differences with Age. *J Allergy Clin Immunol Pract*. 2018;6(2):545-554.e4.
2. Chung KF, et al. International ERS/ATS guidelines on definition, evaluation and treatment of severe asthma. *European Respiratory Journal*. 2014;43(2):343–373.
3. Dunican EM, et al. Mucus plugs in patients with asthma linked to eosinophilia and airflow obstruction. *J Clin Invest*. 2018;128(3):997–1009.
4. Hays SR, et al. Allergen challenge causes inflammation but not goblet cell degranulation in asthmatic subjects. *Journal of Allergy and Clinical Immunology*. 2001;108(5):784–790.
5. Peters MC, et al. Measures of gene expression in sputum cells can identify TH2-high and TH2-low subtypes of asthma. *Journal of Allergy and Clinical Immunology*. 2014;133(2):388-394.e5.
6. Jeffrey DJ, Onishi Y. The Slow Motion of a Cylinder Next to a Plan Wall. *Q J Mech Appl Math*. 1981;34(2):129–137.
7. Hunt AJ, Gittes F, Howard J. The force exerted by a single kinesin molecule against a viscous load. *Biophys J*. 1994;67(2):766–781.
8. Duncan GA, et al. Microstructural alterations of sputum in cystic fibrosis lung disease. *JCI Insight*. 2016;1(18).
9. Matsui H, et al. A physical linkage between cystic fibrosis airway surface dehydration and Pseudomonas aeruginosa biofilms. *Proc Natl Acad Sci U S A*. 2006;103(48):18131–18136.
10. Dobin A, et al. STAR: ultrafast universal RNA-seq aligner. *Bioinformatics*. 2013;29(1):15–21.
11. Robinson MD, McCarthy DJ, Smyth GK. edgeR: a Bioconductor package for differential expression analysis of digital gene expression data. *Bioinformatics*. 2010;26(1):139–140.
12. Love MI, Huber W, Anders S. Moderated estimation of fold change and dispersion for RNA-seq data with DESeq2. *Genome Biol*. 2014;15(12):1–21.
